# Cannabis-Responsive Biomarkers: A Pharmacometabolomics-Based Application to Evaluate the Impact of Medical Cannabis Treatment on Children with Autism Spectrum Disorder

**DOI:** 10.1089/can.2021.0129

**Published:** 2023-02-06

**Authors:** Michael Siani-Rose, Stephany Cox, Bonni Goldstein, Donald Abrams, Myiesha Taylor, Itzhak Kurek

**Affiliations:** Cannformatics, Inc., San Francisco, California, USA.

**Keywords:** medical cannabis, autism, biomarkers, metabolomics, saliva, children

## Abstract

**Introduction:**

Autism spectrum disorder (ASD) is a group of neurodevelopmental conditions that impact behavior, communication, social interaction, and learning abilities. Treatment of ASD with medical cannabis (MC) shows promising results in reducing the severity of certain behavioral aspects. The goals of this observational study are to demonstrate the potential of metabolic biomarkers to (1) objectively determine the impact on metabolites of MC treatment and (2) suggest the metabolic pathways of children with ASD, who respond to MC treatment.

**Materials and Methods:**

The impact of effective physician-supervised MC treatment on children with ASD (*n*=15), compared with an age-matched group of typically developing (TD; *n*=9) children, was evaluated in an observational study design. Each child followed a unique MC regimen determined by their specific response over at least 1 year of treatment, which included the following: tetrahydrocannabinol-dominant MC (dosing range 0.05–50 mg per dose) in 40% of children and cannabidiol-dominant MC (dosing range 7.5–200 mg per dose) in 60% of children. Samples from the ASD group collected pre-MC treatment and at time of maximal impact, and from the TD group, were subjected to salivary metabolomics analysis. Ten minutes before saliva sampling, parents filled out behavioral rating surveys.

**Results:**

Sixty-five potential cannabis-responsive biomarkers exhibiting a shift toward the TD physiological levels were identified in children with ASD after MC treatment. For each biomarker, the physiological levels were determined based on the values detected in the TD group. A similar qualitative improvement trend in children with ASD treated with MC was also observed in the behavioral surveys. Twenty-three potential Cannabis-Responsive biomarkers exhibiting change toward TD mean were categorized as anti-inflammatory, bioenergy associated, neurotransmitters, amino acids, and endocannabinoids. The changes in the levels of the Cannabis-Responsive biomarkers N-acetylaspartic acid, spermine, and dehydroisoandrosterone 3-sulfate have been previously linked to behavioral symptoms commonly observed in individuals with ASD.

**Conclusions:**

Our results suggest Cannabis-Responsive biomarkers shift toward the TD mean after MC treatment and can potentially quantify benefit at the metabolic level. These changes appear to be similar to the trend described in behavior surveys. Larger trials are needed to confirm these preliminary findings.

## Introduction

Autism spectrum disorder (ASD) is a heterogeneous group of neurodevelopmental conditions characterized by deficits in social interaction and communication and restricted, repetitive, and/or stereotyped patterns of behavior.^1^ It is a lifelong condition with onset through a cascade of biological processes or environmental factors such as inflammation or oxidative stress, ultimately leading to a pleiotropic metabolic effect resulting in high heterogeneity in the forms of ASD.^2,3^

ASD prevalence is continuously increasing; as of 2016, 1 in 54 children were diagnosed by age 8 in the U.S. population.^4^ ASD is diagnosed through an extensive evaluation of the child's developmental history, parental reports, and in-person behavioral assessment by a qualified clinician.^5^ Although primarily treated through educational and behavioral services,^6^ 48% of diagnosed children also use prescription drugs, including stimulants, antidepressants, antipsychotics, anticonvulsants, and antianxiety medication to reduce behavioral symptoms such as hyperactivity, irritability, and aggression.^7^

Medical cannabis (MC) shows potential for treating children with ASD. In 2010, Kurz and Blaas^8^ demonstrated the effectiveness of dronabinol (a synthetic form of delta-9-tetrahydrocannabinol [THC]) as a supplementary therapy in a single-case study of a child with ASD. Schleider et al.^9^ showed that MC treatment with 30% cannabidiol (CBD) and 1.5% THC was well tolerated, safe, and effective in children with ASD, with a significant positive impact on quality of life, mood, ability to concentrate, sleep, and performance of daily activities. Low THC and high CBD formulations can also improve behavioral outbursts, anxiety, and communication in children with ASD.^10^

MC treatment for children with ASD is a challenging process for clinicians and families. There is no existing methodology to objectively quantify the impact of MC on the child and current methods require parental involvement, which may affect the behavior of the child and therefore interfere with the outcome. Personalization of treatment based on the analysis of information extracted from metabolite profiles of saliva (pharmacometabolomics) presents an opportunity to maximize treatment efficacy and reduce potential side effects.

Metabolomics, a high-throughput method to evaluate the concentration of metabolites, is routinely used to quantify the response of an individual to physiological or pathophysiological changes.^11^ Metabolic-based biomarkers from urine, plasma, and tissues have emerged as tools for the development of biomarkers for screening and diagnosis of ASD in children.^12^ Furthermore, changes in urinary metabolite levels have been successfully used to demonstrate the potential of metabolomics in conjunction with behavioral observation to elucidate the impact of antioxidants on clinical improvements of children with ASD.^13^

Developing Cannabis-Responsive biomarkers is the first step to objectively quantify the impact of MC treatment. It also presents an opportunity to better understand the mechanism of action of active cannabinoids on symptoms of ASD. To demonstrate the potential of quantified Cannabis-Responsive biomarkers to assess the impact of MC treatment, and to gain insight on the possible mechanism of action, we conducted an observational study of 15 children with ASD, successfully treated with MC.

The objectives of this pilot study were to (1) identify Cannabis-Responsive biomarkers, namely metabolites that change pre- and post-MC treatment; (2) determine if changes in the Cannabis-Responsive biomarkers shift the levels toward the physiological values found in the typically developing (TD) control group; (3) confirm that MC treatment reduces the presence, severity, and/or frequency of social-emotional and/or behavioral difficulties, as reported by parental observation; (4) determine if the Cannabis-Responsive biomarkers are common to all or some of the children with ASD (metabolomics profile); and (5) determine if the Cannabis-Responsive biomarkers suggest mechanism of action.

## Materials and Methods

### Participants

Children with ASD were recruited through Canna-Centers Wellness and Education (Lawndale, CA) or Whole Plant Access for Autism (WPA4A, a 501c3 nonprofit company, Canyon Lake, CA). The inclusion criteria included the following: (1) ASD diagnosed by a qualified medical or behavioral health clinician (e.g., psychologist, psychiatrist, and pediatrician); (2) MC treatment under physician supervision as permitted by California law with signs of improvement based on parental reports; (3) age between 6 and 12 years; and (4) ability to donate saliva without discomfort using the passive drool method and providing up to four samples.

The exclusion criteria were as follows: (1) children who require cannabis more frequently than every 8 h; (2) traumatic brain injury with any known cognitive consequence or loss of consciousness for more than 5 min; and (3) diagnosed with epilepsy.

Age-matched TD control group was recruited through local online parent groups. The inclusion criteria were as follows: (1) age between 6 and 12 years; (2) no individual or immediate family history of established or suspected medical diagnoses and/or developmental disabilities (e.g., autism, attention deficit hyperactivity disorder, intellectual disability, epilepsy, and genetic disorders); and (3) participant has never received special education evaluations and/or services (Individualized Education Program, 504 Plan).

The study protocol was reviewed and approved by Ethical & Independent Review Services, an Association for the Accreditation of Human Research Protection Programs, Inc. (AAHRPP) certified institutional review board (ref 20114-01X). Parents/guardians of participating children signed an informed consent form and TD children from the control group signed an assent form.

### Study design

The impact of MC treatment on children with ASD was studied in an observational study design. To ensure maximal reproducibility of study outcomes, parents of children from both groups were instructed to collect samples in the morning.

In this observational study, all children with ASD were treated with tested MC products available through the CA Medical Marijuana program, as described in Table 1. Children were not treated with MC for at least 8 h before the study (washout period), allowing decay of the previous cannabis treatment, and did not eat and drink foods with high sugar, acidity, and caffeine content at least 1 h before saliva collection. Saliva samples before MC treatment (“PRE”) were collected as follows: (1) mouth rinsing 20 min before saliva collection; (2) completion of brief behavioral survey by the parent 10 min before dose of MC; and (3) saliva collection using Saliva Passive Drool Collection Kit (Salimetrics, LLC, Carlsbad, CA).

**Table 1.** Treatment Characteristics of the Autism Spectrum Disorder Group

Description				Cannabinoid content (mg) per treatment						
ID no.	Age (year)	Gender	Dosage/day (times)	Method	THC	CBD	CBG	CBN	THCA	CBDA
A01	6	Boy	2 (M, N)	Edible	1				17	
A02	7	Boy	3 (M, N, E)	Tincture	10	30		30		
A03	7	Boy	1 (M)	Edible^a^	10		20			
A05	8	Boy	2 (M, N)	Tincture	1		20		15	
A06	8	Boy	2 (M, N)	Tincture	10	35				
A08	9	Boy	2 (M, N)	Edible^a^	5	100	20			
A09	10	Girl	3 (M, N, E)	Tincture	50					75
A11	10	Girl	1 (M)	Tincture	3	50				
A12	11	Boy	1 (M)	Edible^a^		60	50			
A13	11	Boy	2 (M, N)	Edible		85	25			
A14	11	Boy	2 (M, N)	Edible^a^	10	100				
A15	11	Boy	2 (M, N)	Tincture		200				
A16	12	Boy	3 (M, N, E)	Edible^a^	15	7.5				
A17	12	Boy	2 (M, N)	Tincture	4.5	10		3		
A18	12	Boy	2 (M, N)	Tincture	0.05				5	12

^a^ Indicates children treated with tincture together with food, which is considered an edible delivery. MC treatment time morning (M), noon (N), and evening (E) are indicated.

CBD, cannabidiol; CBDA, cannabidiolic acid; CBG, cannabigerol; CBN, cannabinol; MC, medical cannabis; THC, tetrahydrocannabinol; THCA, tetrahydrocannabinolic acid.

Saliva samples post-MC treatment were collected at “PEAK”—approximately 90 min after MC treatment, when treatment was reported by parents as time of maximal impact. For each treatment, sampling procedures were the same as pre-treatment. Similarly, the TD group provided saliva sample in the morning.

### Untargeted metabolomic analysis

#### Sample collection

Saliva samples were collected using the Passive Drool Collection Kit paired with the 2 mL SalivaBio cryovials according to instructions provided by the manufacturer (Salimetrics Carlsbad, CA; Salimetrics LLC; https://salimetrics.com/saliva-collection-handbook).

Immediately after collection, saliva was stored temporarily (up to 24 h) at −20°C, and then at −80°C until mass spectrometry analysis was performed by Human Metabolome Technologies, Inc. (Tsuruoka, Japan).

#### Metabolite analysis

Samples were thawed on ice and divided into two tubes for untargeted metabolomics analysis using the Dual Scan package of Human Metabolome Technologies, Inc. Capillary electrophoresis–time-of-flight-mass spectrometry (CE-TOF-MS) and rapid resolution liquid chromatography–time-of-flight-mass spectrometry (RRLC-TOF-MS) were conducted as follows: (1) CE-TOF-MS-based metabolomics was carried out using the Agilent 7100 Capillary Electrophoresis System (Agilent Technologies, Inc., Santa Clara, CA) and fused silica capillary i.d. 50 μm×80 cm column.

Samples (40 μL and 10 mL Milli-Q water containing 1000 μM internal standards) were mixed and filtered through a 5-kDa cutoff filter (ULTRAFREE-MC-PLHCC, Human Metabolome Technologies, Inc.) to remove macromolecules; and (2) RRLC-TOF-MS-based metabolomics was carried out using Agilent 1200 series RRLC system SL (Agilent Technologies, Inc., Santa Clara, CA) and ODS column, 2×50 mm, 2 μm and (Agilent Technologies, Inc.). Samples (60 μL sample, 40 μL of Milli-Q water, and 300 μL of methanol containing 4 μM internal standards) were centrifuged (2300 *g*, 4°C, 5 min) and the supernatant was desiccated and resuspended in 200 μL of 50% isopropanol and Milli-Q water (v/v) immediately before metabolite analysis.

Peaks detected in the CE-TOF-MS and liquid chromatography–time-of-flight-mass spectrometry (LC-TOF-MS) were analyzed using automatic integration software (MasterHands ver. 2.18.0.1 developed at Keio University).^14^

Putative metabolites were assigned from the Human Metabolome Technologies, Inc., standard library, and Known-Unknown peak library on the basis of m/z and migration time (MT), with tolerance of ±0.5 min in MT and ±mass error of 10 ppm in m/z for CE-TOF-MS, and on the basis of m/z and retention time (RT) with tolerance of ±0.3 min in RT and ±25 ppm for LC-TOF-MS. Putative metabolites were subjected to quality control analysis, including baseline subtraction, dataset normalization, and alignment visualization on two-dimensional plots (m/z and time axis) with matching metabolite standards, and detection of significant differences between metabolites.

### Data analysis

The children with ASD in this study were successfully being treated with MC under physician supervision for at least 1 year, with each taking different cannabinoid content and dosages based on their individual responses as described in Table 1.

Therefore, we considered each child as independent case to demonstrate the presence of potential cannabis-responsive biomarkers in response to different commercially available products, with the limitations of small sample size as reflected in our workflow: (1) identify metabolites that change [(PEAK − PRE)/PRE] using CountPatientDiffUpDowns algorithm; (2) per each metabolite from (1), the impact of MC treatment was binned by the number of standard deviation (SDEV; *z*-scores) against the metabolite's TD control group using the ComparePatientToNeurotypic algorithm.

This algorithm also sorts the metabolites and defines cannabis-responsive biomarkers according to the parameters defined below; (3) *p*-value was calculated using *t*-test on PRE to PEAK values of relative peak area to identify potential cannabis-responsive biomarkers that are common to the cohort of children with ASD.

This methodology allowed us to (1) identify highly abundant potential cannabis-responsive biomarkers covering more than half of the children, the main criterion in developing pharmacodynamics biomarkers, and (2) identify potential cannabis-responsive biomarkers that respond differently to different MC treatments based on the relative change toward the physiological levels determined by the TD control group.

The algorithms developed by Cannformatics, Inc., (San Francisco, CA) are described in detail in Supplementary Data S1. Briefly, (1) CountPatientDiffUpDowns counts the number of ASD children who show a relative increase or decrease in the concentration of particular metabolites after treatment with relevant cannabis product and (2) ComparePatientToNeurotypic calculates the mean (MEAN) relative to the mean and SDEV of the nine TD individuals. The algorithm then bins the ASD subject PRE and PEAK values relative to the TD mean and SDEVs (±0.5 SDEV, ±1.0 SDEV, ±2.0 SDEV, and ±4.0 SDEV) for each individual metabolite. This permits us to calculate the movement (difference) from PRE to PEAK relative to the TD mean for each metabolite.

This effectively determines the direction vector (toward, away, or no movement) relative to the TD mean values for each metabolite. The two algorithms allow us to assess each biomarker according to its abundance (number of children) and impact (*z*-values), essential criteria in defining cannabis-responsive biomarkers.

This setup limited the statistical significance of the cannabis-responsive biomarkers because (1) each child was treated with different cannabinoid combinations that affect differently the outcomes at PRE and (2) we do not know at this stage if every biomarker detected can reach the physiological levels detected in the TD group for the type of ASD.

### Behavioral evaluation

Parents of the ASD group and TD control group completed the following rating forms about their child's social, emotional, and behavioral functioning: (1) adaptive Behavior Assessment System, Third Edition^15^ (ABAS-3); (2) Behavior Assessment System for Children, Third Edition^16^ (BASC-3); and (3) Social Responsiveness Scale, Second Edition^17^ (SRS-2). These rating forms served as a baseline evaluation of typical, daily functioning for subjects over the last several months.

Parents of children in the ASD group also completed a brief survey at time points corresponding to saliva collection (PRE and PEAK). These brief Likert scale surveys captured observational parent report of frequency and/or severity of pre-identified behaviors and/or social-emotional functioning: emotional regulation, behavioral regulation, negative behaviors, restricted/repetitive behaviors, attention, social initiation/response, anxiety, depression/low mood, and adaptive functioning.

## Results

Eighteen children with ASD participated in the sample collection and 15 provided sufficient saliva for untargeted metabolomics analysis. Survey ratings were completed for all 15 children in the ASD group, behavioral rating forms were completed for 14. All nine untreated children in the TD group participated and provided sufficient saliva and completed rating forms. The average age was 9.4 years for the ASD group and 9.3 years for TD group and the ratio of boys:girls was 8:1 in both groups. The MC content, treatment regimen, gender, and ages for the ASD group are described in Table 1.

### Impact of MC treatment on ASD

Parent ratings of symptoms consistent with ASD (SRS-2), indicated all subjects exhibit clinically social impairment (Total Score): 11 were in the severe range, 1 in the moderate range, and 2 in the mild range (Supplementary Data S2). Parent ratings of the TD group (*n*=9) did not endorse clinically significant concerns for attention, anxiety, depression, externalizing behaviors, or atypicality, or for symptoms consistent with ASD. The impact of MC treatment on the behavior of children with ASD was assessed using parent observational surveys completed at PRE and PEAK.

Of those reporting difficulties in the following areas, parents reported improvement in emotional regulation (86.7%); behavioral regulation (86.7%); negative behaviors (i.e., outbursts, tantrums, and aggression; 76.9%); attention (92.6%), and restricted/repetitive behaviors (73.3%) (Supplementary Data S3). Overall, based on parent survey responses, 11 children generally improved, two had mixed response, and two exhibited increased difficulties at PEAK on the day of saliva collection.

### MC treatment shifts the levels of cannabis-responsive biomarkers toward their TD physiological levels

Untargeted metabolomics of PRE and PEAK samples in the ASD and TD groups detected 484 known metabolites consisting of 145 and 339 using RRLC-TOF-MS and time-of-flight-mass spectrometry systems, respectively. CountPatientDiffUpDowns and ComparePatientToNeurotypic analysis of the metabolites identified 65 ASD cannabis-responsive biomarker leads. Since each of these 65 leads was identified in 8–15 children with ASD, we identified a total of 868 data points representing 65 potential ASD cannabis-responsive biomarkers in our dataset. Thirty-one (48%) metabolites were detected in all the participants and 21 (32%) exhibited significant change (*p*<0.05) (Supplementary Data S1).

**FIG. 1.**
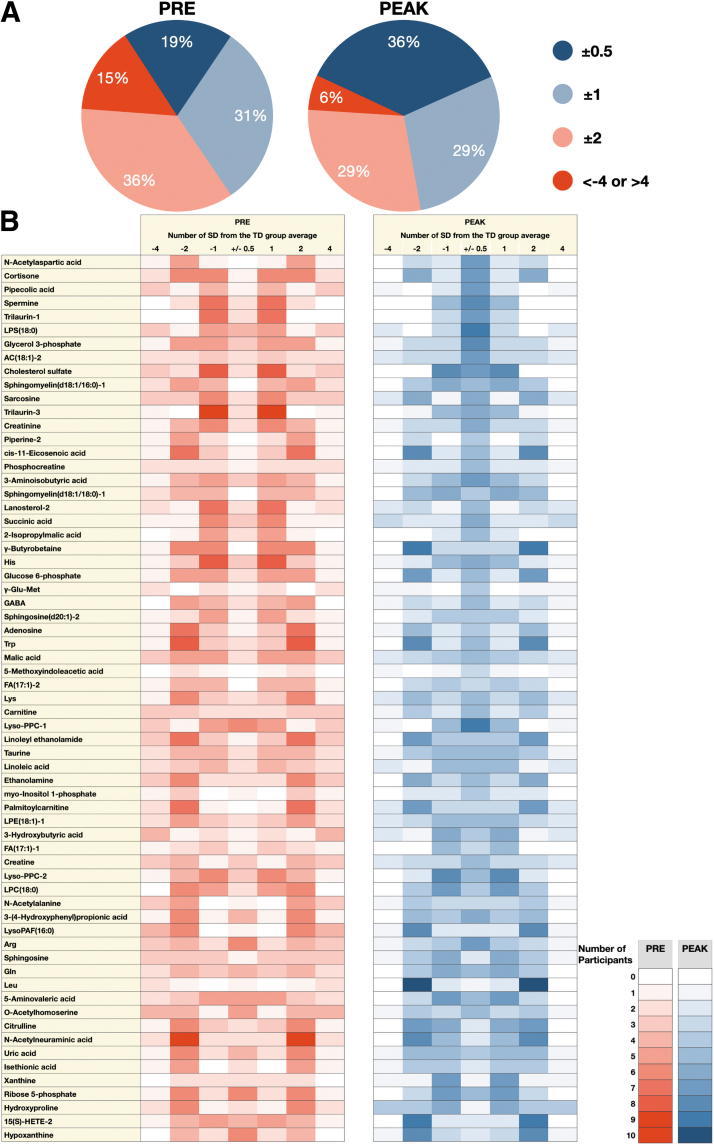
Effect of MC treatment on *z*-score values of potential cannabis-responsive biomarkers: an overview **(A)** and specific **(B)** changes in children with ASD. **(A)** Proportion of *z*-score values pre-treatment and at Peak. Dark blue and light blue are considered within the TD physiological range, while dark red is significantly outside the TD physiological range. **(B)** Pictograph of the number of children for each potential cannabis-responsive biomarker pre-MC treatment (red) and at Peak (blue) time point. The intensity of red and blue represents the number of participants in each range, which is indicated in the right-hand side scales. Dark blue and light blue are considered within the TD physiological range, while dark red is significantly outside the TD physiological range. ASD, autism spectrum disorder; MC, medical cannabis; TD, typically developing. Color images are available online.

As shown in Figure 1A, MC treatment resulted in an increase from 19% at PRE to 36% at PEAK for *z*-scores in ±0.5 SDEV and a decrease from 15% at PRE to 6% at PEAK in the number of metabolites with *z*-score values higher than 4 SDEV and lower than −4 SDEV. In combination, the positive impact of MC treatment affected the levels of potential ASD cannabis-responsive biomarkers by adjusting the levels toward the TD range. All 65 selected metabolites exhibited the trend of shifting the value toward the TD range (±2 SD).

The pictograph in Figure 1B describes the *z*-score value of each potential ASD cannabis-responsive biomarker in children with ASD PRE (red) and PEAK (blue) MC treatment. The intensity of color represents the number of children who lie within the column specified by number of SDEV from the TD mean. Comparison between PRE to PEAK clearly shows that potential cannabis-responsive biomarkers in children with ASD move toward the central column (±0.5 SD) after MC treatment.

This illustrates the potential of MC treatment to drive the potential cannabis-responsive biomarkers toward the physiological level determined by the TD group (TD range). The potential cannabis-responsive biomarkers in Figure 1B were sorted based on the stringency of the tightening toward the TD range.

### Potential cannabis-responsive biomarkers associated with ASD symptoms

N-Acetylaspartic acid (NAA), the highly abundant neurochemical in the brain that correlates with neuronal integrity,^18^ exhibited the highest number of children with ASD moving to the tightest TD range (mean ±0.5 SD).

**FIG. 2.**
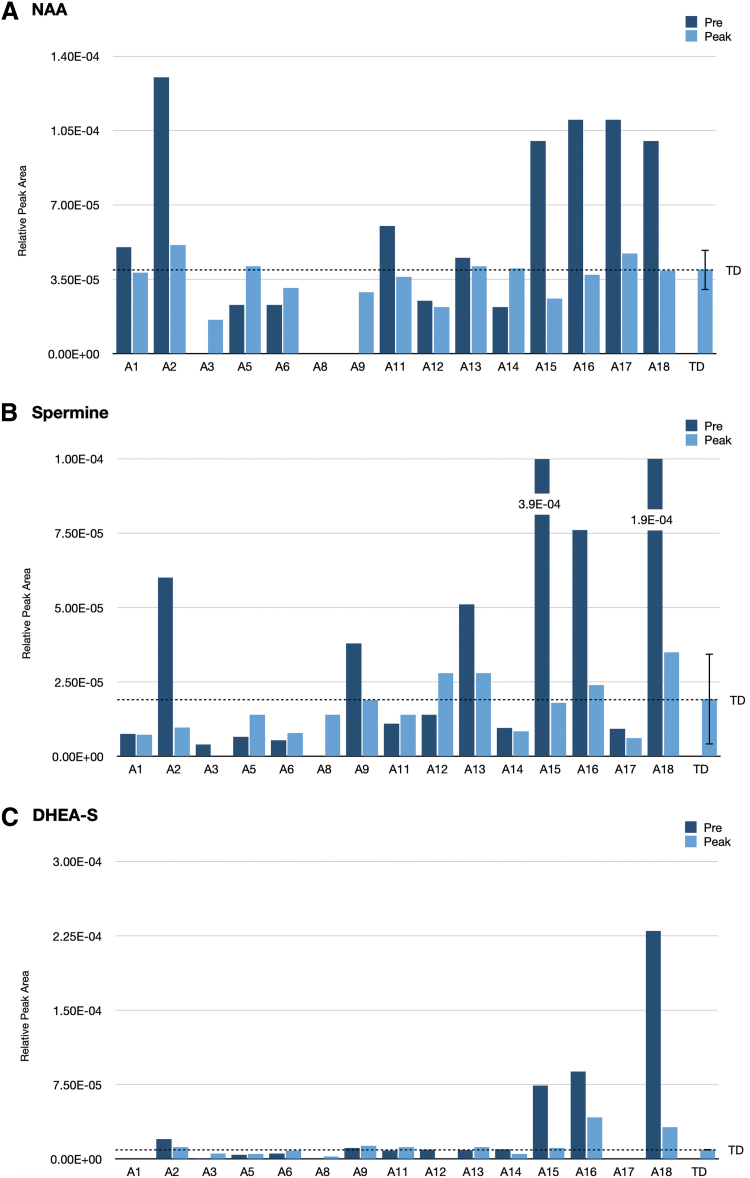
Child-specific quantitation of the specific potential cannabis-responsive biomarkers: **(A)** NAA, **(B)** Spermine, and **(C)** DHEA-S. Children with ASD are sorted by age from age 6 to 12 years. Each child A1–A18 has two bars: dark blue represents concentration before treatment (baseline) and light blue represents concentration after treatment. The average of the TD group with ±SD (dashed line) represents the neurotypic physiologic range. DHEA-S, dehydroepiandrosterone sulfate; NAA, N-Acetylaspartic acid. Color images are available online.

As illustrated in Figure 2A and Table 2, no child with ASD was in the physiological range of ±0.5 SDEV, and only one was in the ±1 SDEV range at PRE. Eight children with ASD were greater than 4 SDEV from the TD range. MC treatment (PEAK) significantly (*p*=0.011) shifted the NAA levels toward the TD physiological range with seven children moving into the ±0.5 SDEV range and two into the ±1 SDEV range, indicating a significant impact of MC on the movement toward the TD mean. Due to the small sample size (*n*=15), we could not determine correlations to age, gender, and cannabinoid content on the impact of NAA levels.

**Table 2.** N-Acetylaspartic Acid Analysis Using the ComparePatientToNeurotypic Algorithm

Bins TD range	No. of ASD children (PRE)	No. of MC-treated ASD children (within range)	Final MC-treated number (PEAK)
Mean±0.5	0	7	7
Mean±1.0	1	9 (2 additional)	2
Mean±2.0	7	12 (3 additional)	3
Mean±04.0	7	12 (0 additional)	0
Mean>4.0	8	3 (reduction)	3
Total	15 Children		15 Children

ASD, autism spectrum disorder; TD, typically developing.

Spermine, the inflammatory pain inducer^19^ found in very high levels in four children (A2, A13, A15, A16, and A18), was reduced from 2 to 24 SDEV above the TD mean at PRE toward ±1 SD from the TD mean at PEAK (*p*=0.05) (Fig. 2B). MC treatment showed a slight increase in subjects A5 and A11 by shifting up spermine *z*-score levels from −1 SDEV to −0.5 SDEV. MC treatment did not change *z*-scores for subjects A1, A6, A14, and A17 at their *z*-score levels. Although, spermine did not exhibit a significant *p*-value, it may be a useful biomarker in a subset of children with ASD who experience pain.

Dehydroepiandrosterone sulfate (DHEA-S) serves as precursors in the adrenal glands to male androgens and female active estrogens. Since high levels of DHEA-S are associated with aggression in psychiatric disorders, we examined these levels in our study.^20,21^ Although DHEA-S did not meet our criteria of change in at least nine children, we found three out of seven, who were 11–12 years old (A15, A16, and A18), exhibited very high levels, suggesting an association of DHEA-S with age (Fig. 2C). DHEA-S levels were reduced at PEAK, but remain higher than the TD group.

### Other potential cannabis-responsive biomarkers

**FIG. 3.**
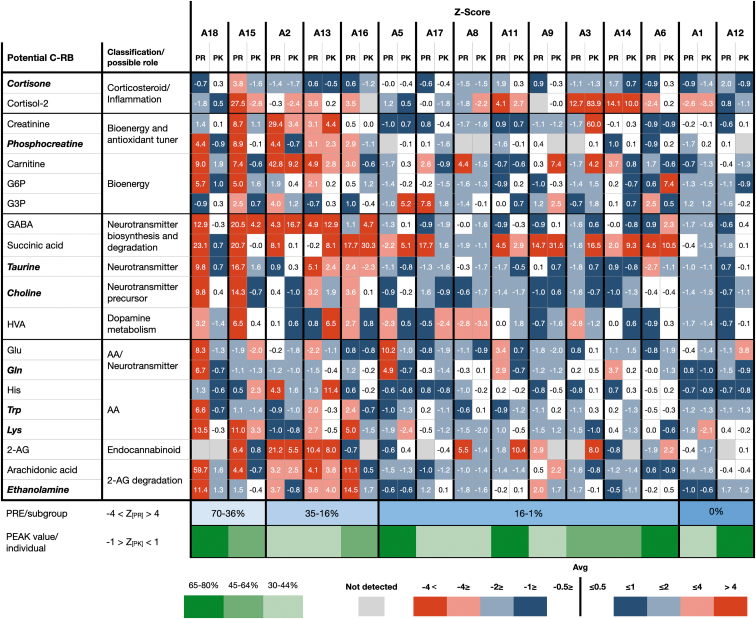
Pictograph of metabolic profile of potential cannabis-responsive biomarker color-coded levels in response to MC treatment represented by *z*-score values at PRE (PR) and PEAK (PK) time points in children with ASD. Significant impact (*p*≤0.05) of MC on potential cannabis-responsive biomarkers is indicated in bold italic. Color images are available online.

The main potential cannabis-responsive biomarkers exhibiting major or significant changes (*p*≤0.05 in bold, italic) include metabolites with roles in inflammation, bioenergetics, amino acid metabolism, neuronal activity, and the endocannabinoid system (Fig. 3). An additional large group of lipids will be described elsewhere (Siani-Rose et al., article in preparation).

Figure 3 highlights the potential cannabis-responsive biomarkers exhibiting the largest shifts toward TD mean at PEAK. Since this study did not determine the hierarchy among the potential cannabis-responsive biomarkers, we counted the number of *z*-scores in each category (>±4 SDEV; <±4 SDEV; >±2 SDEV; <±2 SDEV; >±1 SDEV; <±1 SDEV; >±0.5 SDEV; and <±0.5 SDEV). There appear to be four subgroups (color coded) based on the high *z*-score values identified at PRE (A18 and A15), (A2, A13, and A16), (A5, A17, A8, A11, A9, A3, A14, and A6), and (A1 and A12). The total number of potential cannabis-responsive biomarkers exhibiting TD physiological levels (Z[Pk]≤±1) at PEAK may evaluate the impact of specific MC treatments.

With the limitations described below, MC treatment exhibited a high impact across different suggested groups/forms of ASD (A18, A5, A11, A6, and A12). Multiple comparisons did not show statistical difference between these small groups in which only eight potential biomarkers exhibit *p*-values lower than 0.05.

## Discussion

There is growing evidence that MC can successfully alleviate behavioral symptoms of children with ASD.^22^ The lack of objectively quantified data regarding effectiveness, safety, and mechanism of action makes it difficult to determine the effectiveness of the treatment.^23^ For example, current methods do not characterize the pathophysiology of inflammation, pain, cellular energy, and neurotransmitter regulation.

Metabolic biomarkers offer an attractive method to quantify, stratify, and personalize the MC treatment for individuals with ASD. The quantitative data accumulated from biomarkers to form metabolic profiles, known as pharmacometabolomics, is an emerging approach to personalized medicine.^11^ The goals of this study were to demonstrate the potential capabilities of a new class of metabolites, cannabis-responsive biomarkers, to objectively assess the impact of MC treatment and to investigate the metabolic pathways affected by MC treatment. To focus solely on these goals, we chose children with ASD who are successfully being treated with MC supervised by medical doctors.

A biomarker is “a defined characteristic that is measured as an indicator of normal biological processes, pathogenic processes, or responses to an exposure or intervention, including therapeutic interventions,”^24^ which can be categorized by subtype such as diagnostic, monitoring, pharmacodynamic/response, and predictive.^25^ All the 65 potential cannabis-responsive biomarkers identified in this study are pharmacodynamic/response biomarkers whose levels change in response to MC treatment and may be extremely important for clinical evaluation and development of new treatment. In addition, NAA, spermine, DHEA-S, and cortisone found in this group may also be associated with clinical symptom(s)/phenotype.

NAA, a key metabolite found ubiquitously in neuronal cells, is considered a specific biomarker for neuronal number and viability,^26^ with roles in neuronal metabolism, integrity, and energy.^27^ The PRE NAA levels revealed two distinct groups that have either significantly higher (>3 SDEV) or just below/in the range of the TD (±1 SDEV) (Fig. 2A). We observed a striking modulation of NAA levels toward TD levels at PEAK, where treatment reduced the levels in the first group and increased in the second, suggesting stabilization of NAA levels. A previous study suggested that significant reduction of NAA levels, detected by functional magnetic resonance imaging, in the left frontal cortex compared to high-functioning controls may reflect early brain growth dysregulation and ongoing neuroinflammatory processes.^18^

The study also indicated that reduced levels of NAA in ASD were related to neurofunctional abnormalities. While Kleinhans et al.^18^ presented neuron-focused measurements, we detected NAA levels in saliva, which represent systemic levels and most likely show different trends. Although most studies reported low levels of NAA in individuals with ASD, a significantly higher pre-frontal lobe concentration of NAA has been reported in subjects with Asperger syndrome.^28^

Spermine, a major central nervous system polyamine, was shown to induce nociceptive pain through the transient receptor potential vanilloid subtype 1 (TRPV1) receptor in mice under inflammatory conditions with a twofold higher potency than the polyamine putrescine.^29^ High spermine levels of 2–24 SDEV above the TD mean were detected in six children with ASD (A2, A13, A15, A16, and A18), and two of them (A15 and A18) also exhibited high levels of putrescine (3 SDEV and 6 SDEV above the TD mean, respectively) (data not shown).

Both spermine and putrescine levels were shifted toward the TD mean levels at PEAK, and may suggest an anti-hyperalgesic response to CBD and cannabidiolic acid (CBDA). Although CBD and CBDA are known agonists of TRPV1, it has been suggested that CBD can potentially inhibit or desensitize TRPV1 signaling, resulting in an antihyperalgesic response.^30^

The gamma aminobutyric acid (GABA) receptor A (GABA_A_) agonist DHEA-S, which plays an important role in aggressive behavior in avian and mammalian species,^21^ was found in high levels in saliva of children with ASD.^31^ In our study, subjects A15, A16, and A18 showed high salivary DHEA-S levels at PRE, which were reduced at PEAK toward the TD levels.

NAA, DHEA-S, and spermine are reported to have either neuroprotective or antioxidative properties.^32,33^ When compared to the TD group, subjects A15, A16, and A18 (boys ages 11–12 years) were found to exhibit significantly elevated PRE levels of all three potential cannabis-responsive biomarkers: NAA: 7–8 SDEV; spermine: 4–24 SDEV; and DHEA-S: 16–51 SDEV. The levels detected at PEAK represent a decrease of 9- to 14-fold in NAA, 12- to 124-fold in spermine, and 2- to 8-fold in DHEA-S, indicating that MC treatment, specifically CBD and CBDA, has neuroprotective and/or antioxidative actions.^34,35^

A notable change in the levels of ethanolamine and a trend in GABA were detected in response to MC treatment (Fig. 3). 2-Arachidonoylglycerol, arachidonic acid, oleoylethanolamide, palmitoylethanolamide, and linoleoyl ethanolamide were detected, but not impacted by MC treatment (data not shown). Since endocannabinoids are generated on demand and any excess is quickly inactivated and degraded, it is possible that saliva samples collected about 90 min post-MC treatment (PEAK) are not the correct time point and biofluid to evaluate endocannabinoids that are continuously regulated in the brain.

We also found a tightly regulated Glu:Gln ratio with a slight decrease in most of the children (Supplementary Data S4), which was also inconsistent with the ratio reported previously in plasma of children with ASD.^36^

### Limitations

Although we identified some potential cannabis-responsive biomarkers that exhibit significant changes in both PRE and PEAK by shifting the levels toward the TD mean values, we must consider several limitations in this study.

First, the small sample size of children successfully treated with MC in this study did not cover the heterogenicity of the ASD population and cannot indicate that MC provides a solution for all clinical phenotypes of ASD, nor does it show a shift of all biomarkers toward the TD physiological levels. Also, this small sample size did not allow us to associate the content of cannabinoids to the biomarker responses. Second, we conducted an observational study where children with ASD were treated with off-the-shelf MC. Some doses were measured using a dropper, which may not be accurate. Third, we gave equal impact to all the potential cannabis-responsive biomarkers.

As we continue to grow our database, it will be possible to stratify and focus on specific biomarkers related to the metabolic pathways affected by ASD and the clinical phenotype. Fourth, since we used saliva as the biofluid, we cannot rule out that the biomarker changes observed are not in their physiological context. Fifth, since a child's behavior is influenced by environmental factors and varies from day to day, our saliva samples and surveys may not represent the full range of behaviors for each child.

## Conclusions

Taken collectively, the data presented demonstrate the potential of pharmacometabolomics to identify metabolic biomarkers that respond to MC treatment, which may be used to construct metabolic profiles for future personalization of MC treatment in children with ASD. This is the first study to define salivary ASD Cannabis-Responsive biomarkers as measurable metabolites found in saliva of children with ASD that change in response to MC and can objectively quantify the impact of the treatment. These biomarkers may assist in diagnosis, discovery of therapeutic mechanisms of action, and influence treatment of ASD in the future.

Cannabis-responsive biomarkers can be expanded beyond ASD to any medical condition treated by at least one cannabinoid. As cannabis-responsive metabolic profiles are collected and developed, we hope to predict the optimal cannabinoid profile and dosage for each individual based on a pre-dose sample.

## Supplementary Material

Supplemental data

Supplemental data

Supplemental data

Supplemental data

## Abbreviations Used

ASD: autism spectrum disorder
CBD: cannabidiol
CBDA: cannabidiolic acid
CBG: cannabigerol
CBN: cannabinol
CE-TOF-MS: capillary electrophoresis–time-of-flight-mass spectrometry
DHEA-S: dehydroepiandrosterone sulfate
GABA: gamma aminobutyric acid
LC-TOF-MS: liquid chromatography–time-of-flight-mass spectrometry
MC: medical cannabis
MT: migration time
NAA: N-acetylaspartic acid
RRLC-TOF-MS: Rapid resolution liquid chromatography–time-of-flight-mass spectrometry
RT: retention time
SDEV: standard deviation of the mean
SRS-2: social responsiveness scale, second edition
TD: typically developing
THC: tetrahydrocannabinol
THCA: tetrahydrocannabinolic acid
TRPV1: transient receptor potential vanilloid subtype 1
