# Supplementary material for: Cannabis-Responsive Biomarkers: A Pharmacometabolomics-Based Application to Evaluate the Impact of Medical Cannabis Treatment on Children with Autism Spectrum Disorder
Source: Cannabis Cannabinoid Res. 2023 Feb 6;8(1):126–37. doi: 10.1089/can.2021.0129 (PMC9940806; doi:10.1089/can.2021.0129)

**S1: Supplementary Material Data Analysis**

CountPatientDiffUpDowns algorithm

This algorithm calculates the relative change in concentration for each patient between pre-treatment (PRE) and Peak post-treatment (PEAK). For each metabolite, and for each patient, the algorithm calculates ((PEAK - PRE) / PRE), when both PRE and PEAK values are above the non-detect (N.D.) limit. To develop a valid biomarker, a metabolite must be detected in most of the children (≥8). Therefore N.D. (not detected) values were not statistically manipulated and counted. Then for each metabolite, the number of children showing a decrease is counted. Likewise, the number of children showing an increase is calculated. A percent of total for both the increased and decreased is calculated. We then kept only metabolites with greater than or equal to 10 children with valid results. Any metabolites with less than 7 children with valid results were removed from consideration. This algorithm allowed for sorting of the metabolites based on these percentages. Using a 70% cutoff, we assigned a label of "Majority Decrease" or "Majority Increase". This algorithm also pre-processes ASD patient data for comparison to TD individuals in the next step.

ComparePatientToNeurotypic Algorithm

This method calculates the Z-score, namely, mean (MEAN) and standard deviation (SDEV) across ten TD individuals (baseline only, no treatment). The TD individuals are in the same age range as the 15 ASD subjects. The algorithm then performs a calculation of the movement (from PRE to PEAK) either towards, away or no movement relative to the TD mean values for each patient and each metabolite. This directional movement is calculated by binning values, based on number of SDEVs (+/- 0.5 SDEV, 1.0 SDEV, 2.0 SDEV, 4.0 SDEV) from the TD mean, for each person for each metabolite before and after treatment.

For each metabolite, we calculate the mean and SDEV across the TD group with values greater than non-detect (N.D.). The MEAN and +/- 1.0 SDEV represents the TD range. The ranges of 0.5, 1.0, 2.0 and 4.0 standard deviations around the mean represent buckets of decreasing tightness or stringency.

We calculated the number of ASD children that fall into each of these bins, for both PRE and PEAK samples. Additionally, we then calculate the change across these buckets from PRE to POST. For example, the difference of PEAK number of children vs. PRE number of children within 0.5 SDEV of the TD mean. When this difference increases, we know that children who have responded to the treatment have moved towards the TD mean.

The total number of metabolites with a biochemical stringency of a z- score greater than 2 or less than −2 score > in ≥ 30 % of children with ASD indicated a significant change from the TD physiological values (Kerkhofs et al., 2020).

Table 1 complements Figure 1B and indicates the metabolite name, abundance (number of children it presents in) and the significance (p values) of the MC treatment on the metabolite changes. Metabolites in italics will be discussed elsewhere.


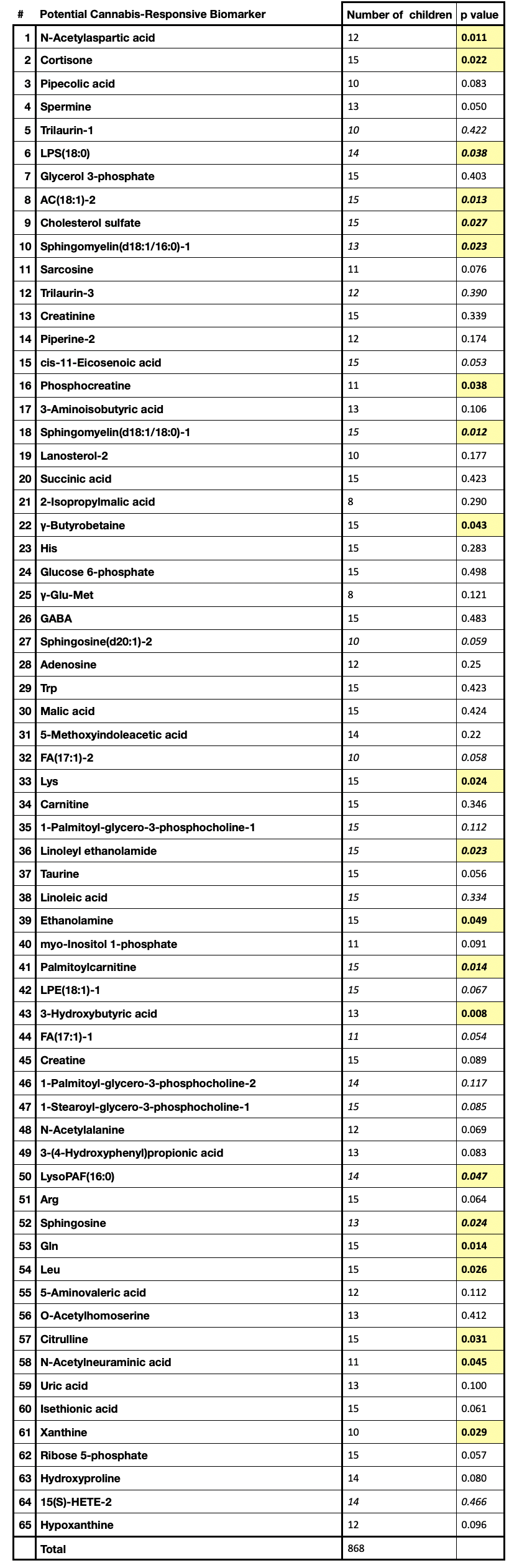

Supplement: Supplemental data [file Supp_DataS1.docx]
