# Supplementary material for: Cannabis-Responsive Biomarkers: A Pharmacometabolomics-Based Application to Evaluate the Impact of Medical Cannabis Treatment on Children with Autism Spectrum Disorder
Source: Cannabis Cannabinoid Res. 2023 Feb 6;8(1):126–37. doi: 10.1089/can.2021.0129 (PMC9940806; doi:10.1089/can.2021.0129)
Supplement: Supplemental data [file Supp_DataS2.docx]

**S2: Supplementary Behavioral Ratings**

Across the ASD group, parents also endorsed elevated levels of concerns for behavioral functioning (BASC-3), including hyperactivity (clinically significant n=7; at-risk n=2), attention (clinically significant n=5; at-risk n=2), and atypicality (clinically significant n=11). All subjects were rated to have a moderate (n=3) or severe (n=11) level of restricted and repetitive interests and behaviors (SRS-2). A minority of subjects (n=4, 28.4%) were rated to have clinically significant concerns for aggression, anxiety, and/or depression. Overall, the ASD group had significant impairment in adaptive/daily living skills with 64.3% (n=9) in the Extremely Low Range (ABAS GAC <70), 21.4% (n=3) in the Low range, and 7.1% in both the Below Average (n=1) and Average (n=1) range.


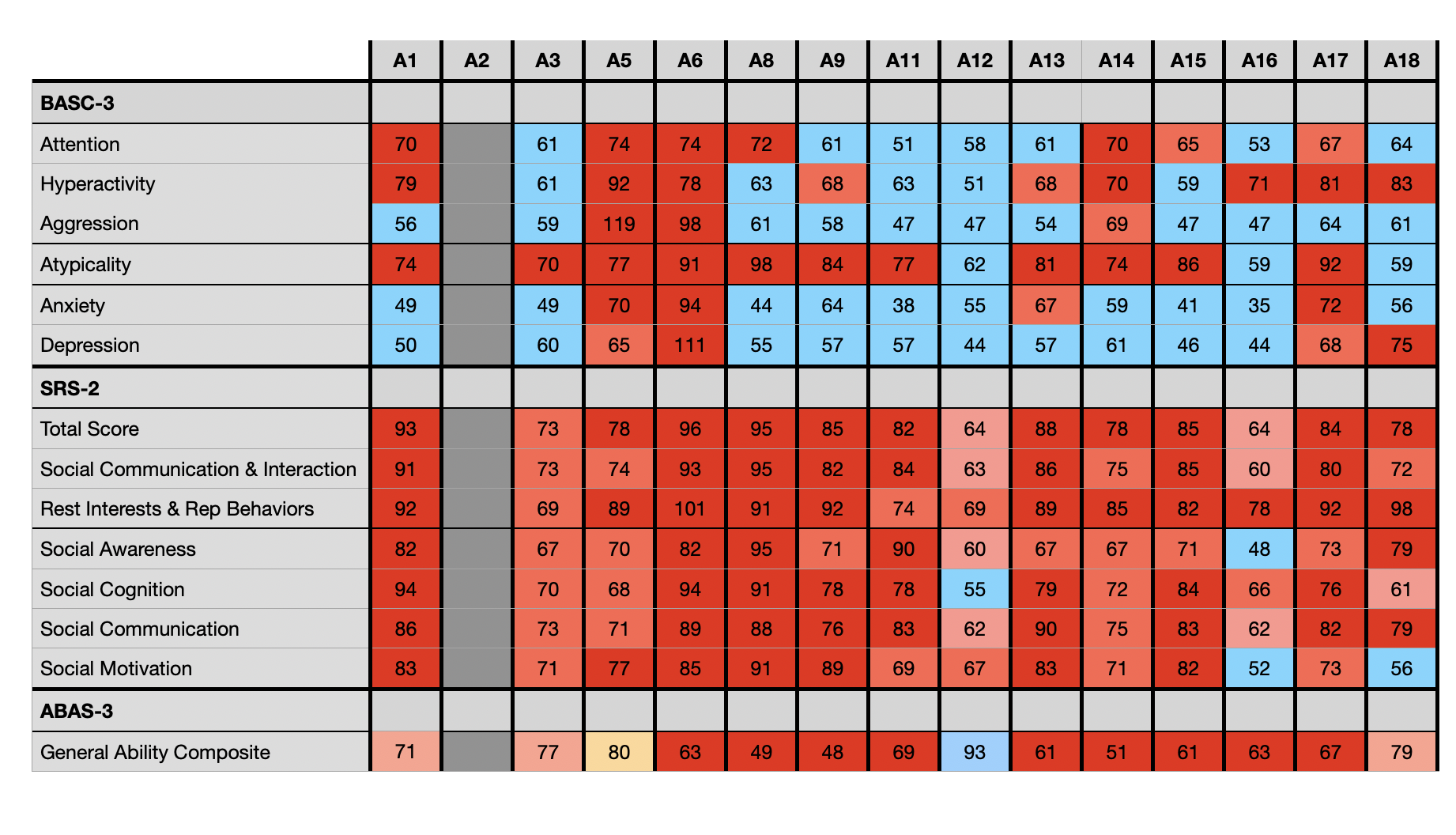


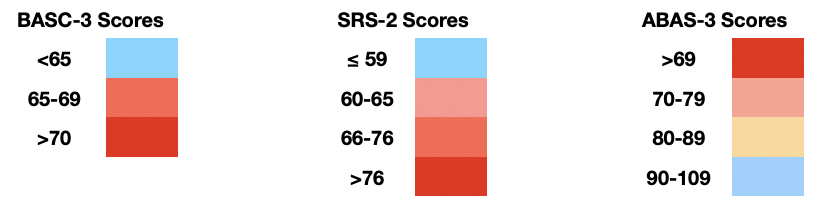


Table S2

BASC-3 and SRS-2, T-scores: Higher scores indicate higher levels of reported problems. For the BASC-3, scores below 65 are considered within normal limits, 65 to 69 mildly elevated/“At-Risk”areas, and above 70 clinically significantly elevated areas of concern. For the SRS-2, scores below 60 are considered within normal limits, 60 to 65 are in the mild range, 66 to 75 are in the moderate range, and above 76 are in the severe range.

ABAS-3, Standard Score: Lower scores indicate higher level of impairment. Scores below 70 are Extremely Low, 70 to 79 are Low, 80 to 89 are Low Average, 90 to 109 are Average.
