# Supplementary material for: Cannabis-Responsive Biomarkers: A Pharmacometabolomics-Based Application to Evaluate the Impact of Medical Cannabis Treatment on Children with Autism Spectrum Disorder
Source: Cannabis Cannabinoid Res. 2023 Feb 6;8(1):126–37. doi: 10.1089/can.2021.0129 (PMC9940806; doi:10.1089/can.2021.0129)

**S3: Supplementary Surveys**

Overall, within the ASD group, there were mild concerns regarding internalizing symptoms (anxiety, low mood) on baseline survey rating forms. Of those reporting symptoms of anxiety (n=10), all reported improvement at PEAK. Of those reporting concern for low mood (sadness, tearfulness; n=9) there was improvement observed in all but one subject, who reported no change. There was not a clear trend of change reported for social skills (interest/motivation, initiation, response) or ability to complete daily tasks independently (adaptive/daily living skills). Overall, based on parent survey responses, 11 children generally improved, two had mixed response, and two exhibited increased difficulties at PEAK.


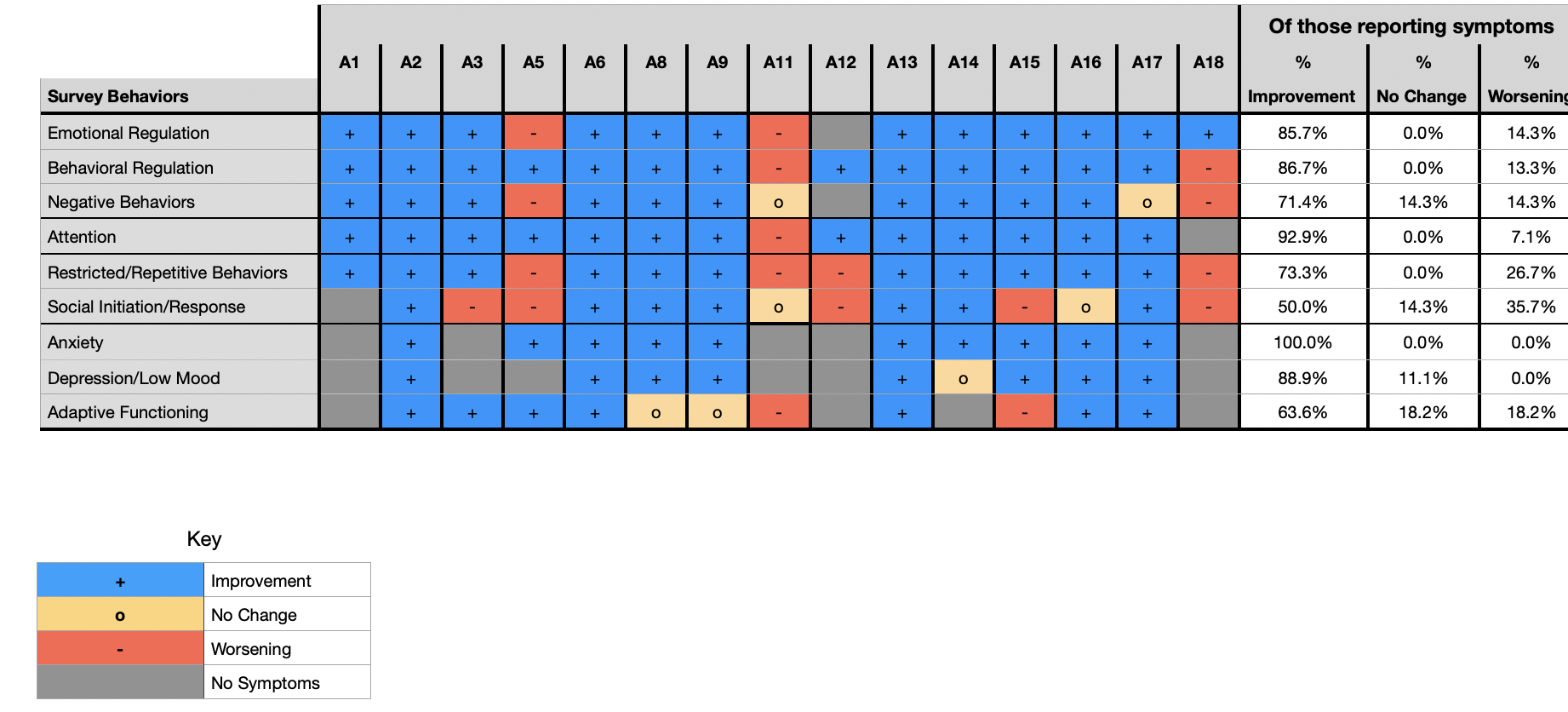

Supplement: Supplemental data [file Supp_DataS3.docx]
