# Supplementary material for: Cannabis-Responsive Biomarkers: A Pharmacometabolomics-Based Application to Evaluate the Impact of Medical Cannabis Treatment on Children with Autism Spectrum Disorder
Source: Cannabis Cannabinoid Res. 2023 Feb 6;8(1):126–37. doi: 10.1089/can.2021.0129 (PMC9940806; doi:10.1089/can.2021.0129)
Supplement: Supplemental data [file Supp_DataS4.docx]

**S4: Supplementary Material Neurotransmitters**

**
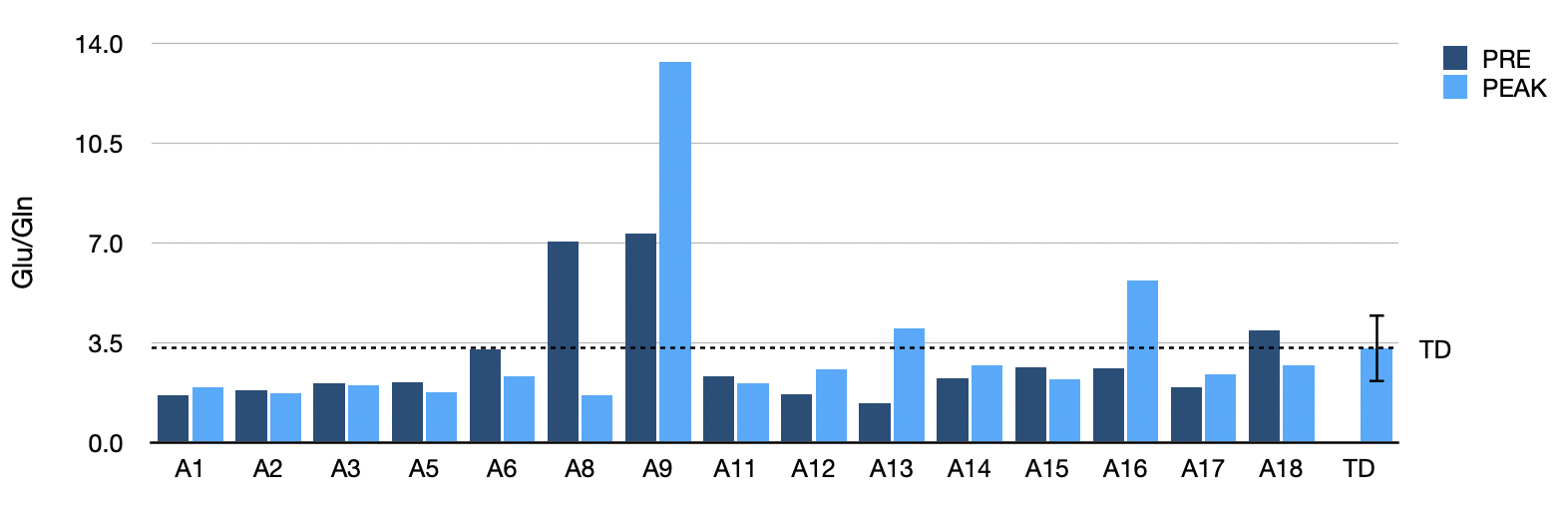
**

Impact of medical cannabis treatment on the ratio of the potential Cannabis-Responsive Biomarkers Glu and Gln. Children with ASD are sorted by age from age 6 - 12. Each child A1-A18 has two bars (dark blue represents Glu/Gln ratio before treatment (baseline) and after treatment (light blue). The average ratio of the TD group (dashed line) with ± SDEV represents the physiologic ratio.
